# Dynamics of DNA methylation and Histone H4 acetylation during floral bud differentiation in azalea

**DOI:** 10.1186/1471-2229-10-10

**Published:** 2010-01-12

**Authors:** Mónica Meijón, Isabel Feito, Luis Valledor, Roberto Rodríguez, María Jesús Cañal

**Affiliations:** 1 Laboratorio de Fisiología Vegetal, Dpto. B.O.S., Facultad de Biología, Universidad de Oviedo, C/Cat. Rodrigo Uria s/n, E-33071, Oviedo, Asturias, Spain; 2 Instituto de Biotecnología de Asturias, (Associated to CSIC) Edificio Santiago Gascón, C/Fernando Bongera s/n, E-33006 Oviedo, Asturias, Spain; 3 SERIDA, Servicio Regional de Investigación Desarrollo Agroalimentario, Finca "La Mata", Apdo 13, E-33820 Grado, Asturias, Spain

## Abstract

**Background:**

The ability to control the timing of flowering is a key strategy for planning production in ornamental species such as azalea, however it requires a thorough understanding of floral transition. Floral transition is achieved through a complex genetic network and regulated by multiple environmental and endogenous cues. Dynamic changes between chromatin states facilitating or inhibiting DNA transcription regulate the expression of floral induction pathways in response to environmental and developmental signals. DNA methylation and histone modifications are involved in controlling the functional state of chromatin and gene expression.

**Results:**

The results of this work indicate that epigenetic mechanisms such as DNA methylation and histone H4 acetylation have opposite and particular dynamics during the transition from vegetative to reproductive development in the apical shoots of azalea. Global levels of DNA methylation and histone H4 acetylation as well as immunodetection of 5-mdC and acetylated H4, in addition to a morphological study have permitted the delimitation of four basic phases in the development of the azalea bud and allowed the identification of a stage of epigenetic reprogramming which showed a sharp decrease of whole DNA methylation similar to that is defined in other developmental processes in plants and in mammals.

**Conclusion:**

The epigenetic control and reorganization of chromatin seem to be decisive for coordinating floral development in azalea. DNA methylation and H4 deacetylation act simultaneously and co-ordinately, restructuring the chromatin and regulating the gene expression during soot apical meristem development and floral differentiation.

## Background

In the ornamental plant industry, azalea production represents a cultivation in expansion. For commercial purposes azalea plants must be compact and well branched [1], although floral quality is the fundamental trait for the commercial success of these species. The flowering promotion, by both increasing the number of flowers and advancing the time of flowering, as well as creating novelty in the flower structure, are major desirable traits in ornamental plant breeding.

The annual cycle of azalea japonica in Asturias (Spain, Europe) involves a period of vegetative active growth from May to September, followed by an apparent rest from October to January, months in which well formed buds are visible. As from January, the floral buds are developed, culminating in March with full bloom. Authors such as Bodson [2] defined four phenological stages in the flower development of azalea:1) transition of the apex from the vegetative to floral condition; 2) development of the inflorescence; 3) dormancy of the inflorescence bud; and 4) opening of flowers after breaking of dormancy.

Transition from the vegetative to reproductive stage is the most dramatic change during plant development, involving the transmission of the integrated signal of floral induction to the floral meristem identity genes and floral morphogenesis. The regulation of this process is essential for plant development, and it is achieved by a complex genetic network. Four major flowering pathways have been characterized in *Arabidopsis*, including environmental induction through photoperiod and temperature, autonomous floral initiation and regulation by gibberellins [3-5]. The switch to flowering involves the integration and coordination of the perception of environment (day length, light conditions and temperature) with endogenous factors such as developmental status and age. This coordination is decisive for reproductive success in plants and its correlation with epigenetic mechanisms such as DNA methylation and histone modification has been demonstrated in several species [4,6-8].

Transitions between different developmental phases in Shoot Apical Meristem (SAM) from vegetative to reproductive stages involve changes in the pattern of cellular differentiation and organ formation. During the vegetative development of the plant, SAM cells are organized at different levels, the central cells remain pluripotent while cells in the periphery contribute to organ formation and eventually differentiate [9,10] but during the floral transition, the cells of the central zone start to divide at a high frequency and the zonation is lost [11]. These changes in the SAM are genetically regulated among other processes by epigenetic mechanisms [8,11-13].

There is increasing evidence that chromatin remodelling is involved in numerous processes of development and differentiation in both plants and animals [14,15]. DNA methylation and histone modification have been revealed as hallmarks that establish the functional status of chromatin domains [16] and confer the flexibility of transcriptional regulation necessary for plant development and adaptive responses to the environment [17-19].

Epigenetic control plays an essential role in the process of cellular differentiation allowing cells to be reprogrammed in order to generate new differentiation pathways [20,21]. DNA methylation, histone modifications and specific chromosomal proteins are involved in controlling the functional state of chromatin and gene expression [14,22,23].

Histone acetylation and DNA methylation can activate or repress transcription by generating "open" or "closed" chromatin configurations, respectively. Generally open chromatin increases the accessibility of the genome to transcription machinery, while closed chromatin represses gene expression by limiting the accessibility [14,22,24]. The acetylation of specific lysine residues within the amino-terminal tails of H3 and H4 and DNA demethylation are positively correlated with open chromatin configuration and gene activity in meristematic tissue of plants [25] and both epigenetic processes are connected by particular molecular routes [22].

The regulation of the *FLOWERING LOCUS C (FLC) *in *Arabidopsis *provides a plant model of how chromatin-modifying systems have emerged as important components in the control of transition to flowering. Genetic and molecular studies have revealed three systems of *FLC *regulation: vernalization, the autonomous pathway, and *FRIGIDA (FRI)*. All these involve changes in the state of *FLC *chromatin by DNA methylation and/or histone acetylation [6,26,27]. *FLC *encodes a repressor of flowering which is a key gene during floral transition in autonomous and vernalization pathways.

Dynamic changes between chromatin states that facilitate or inhibit DNA transcription are important in the transcriptional regulation of floral induction pathways in response to environmental and developmental signals in *Arabidopsis*. The rational manipulation of commercially interesting processes such as the timing of flowering in ornamental species such as azalea requires a thorough understanding of floral transition.

To test the hypothesis that the epigenetic code could be related to floral induction and differentiation in azalea, whole DNA methylation and acetylated histone H4 (AcH4) levels were monitored during floral transition. In addition the spatial distribution of these modifications was followed by immunodetection in order to validate the quantification results and analyze the dynamics of cell differentiation during floral transition in SAM.

## Results

### Histological study and DNA methylation

**Figure 1:**
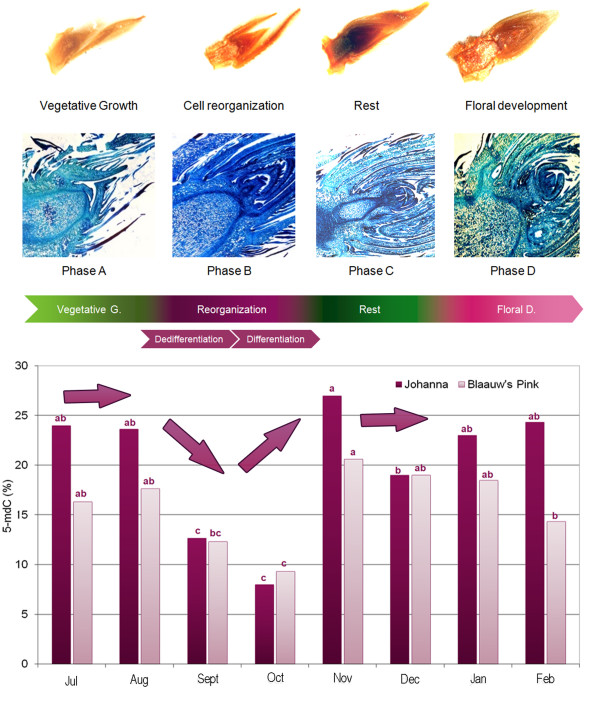
**Profile of DNA methylation (5-mdC %) from July to February in buds of Blaauw's Pink and Johanna cultivars and relationship with histological study of floral bud development**. Within each cultivar, different letters indicate significant differences between dates (Two-way ANOVA; p ≤ 0.001; n = 4).

Macromorphological and micromorphological observations (histological study) carried out in buds collected during floral transition showed differential morphologies and structures (Fig. 1). However it was not possible to define well every phase of floral bud development until DNA methylation analysis was performed.

In both cultivars analyzed the levels of 5- mdC showed similar patterns during the transition period from vegetative to floral bud, although they were significantly higher in the cultivar Johanna in most of the periods sampled (Fig. 1).

DNA methylation was stable during the summer months, July and August. After that, from September to October, a sharp decrease in global DNA methylation took place; followed by a rapid increase, between October and November. In December, both cultivars retained the same level of DNA methylation, 19%. Finally, from January the cultivars showed a slightly different behavior, while in Johanna levels of 5-mdC tended to stabilize, in Blaauw's Pink a decline was observed.

Based on the methylation profiles and the morphology observed it was possible to define four phases or stages in the differentiation and development of the bud: a vegetative phase from July to August, which we named phase A (Fig. 1). At this stage we could see the morphology and structure of a typical vegetative shoot apical meristem with leaf primordia and the meristematic apex. Then a transition phase, from September to November, could be defined taking the methylation as a base, in which initially a sharp decrease in the level of methylation was shown followed by a subsequent hypermethylation (Fig. 1) and named phase B or the stage of cell reorganization. Subsequently, phenologically and at the level of DNA methylation it was possible to observe a period of rest that we have defined as phase C, and it corresponded to the months of December to January. Finally, phase D was named the period from January to March, the stage at which the development of flower bud is completed.

### 5-mdC immunolocation during floral development

**Figure 2:**
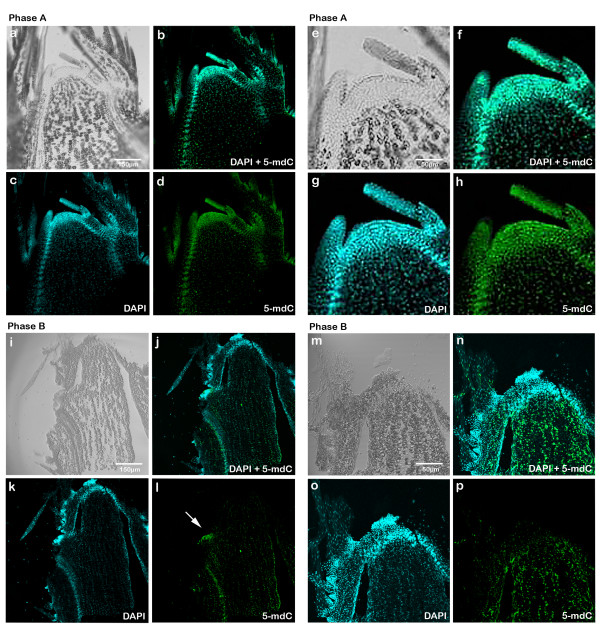
**Immunodetection of 5-mdC in sections of buds in phase A and B of development in longitudinal axis using a confocal microscope**. **(a) **Differential Interference Contrast (DIC) of bud in phase A (10×). **(b) **DAPI (blue signals) superposition and 5-mdC (green signals) of bud in phase A (10×). **(c) **DAPI labelling of nuclei of bud in phase A (10×). **(d) **5-mdC labelling of bud in phase A (10×). **(e) **DIC of bud in phase A (40×). **(f) **DAPI (blue signals) superposition and 5-mdC (green signals) of bud in phase A (40×). **(g) **DAPI labelling of nuclei of bud in phase A (40×). **(h) **5-mdC labelling of bud in phase A (40×). **(i) **DIC of bud in phase B (10×). **(j) **DAPI (blue signals) superposition and 5-mdC (green signals) of bud in phase B (10×). **(k) **DAPI labelling of nuclei of bud in phase B (10×). **(l) **5-mdC labelling of bud in phase B (10×). **(m) **DIC of bud in phase B (20×). **(n) **DAPI (blue signals) superposition and 5-mdC (green signals) of bud in phase B (20×). **(o) **DAPI labelling of nuclei of bud in phase B (20×). **(p) **5-mdC labelling of bud in phase B (20×).

**Figure 3:**
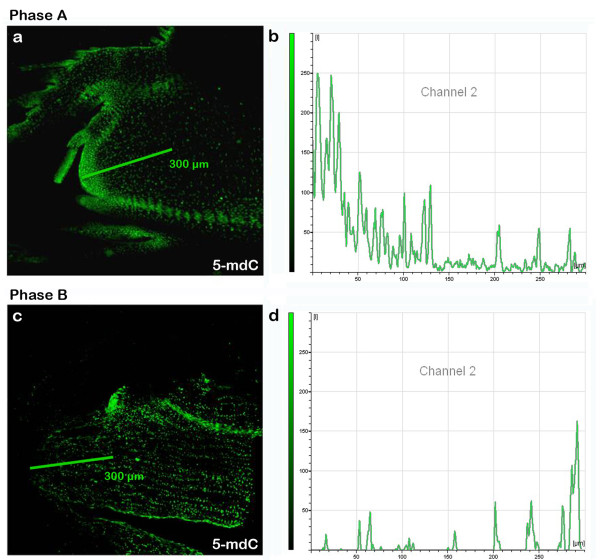
**Intensity of 5-mdC fluorescence markers of buds in phase A and phase B**. **(a) **5-mdC labelling of bud in phase A (10×). **(b) **Intensity of 5-mdC fluorescence markers along the line shown of bud in phase A **(c) **5-mdC labelling of bud in phase B (10×). **(d) **Intensity of 5-mdC fluorescence markers along the line shown of bud in phase B.

In vegetative buds (phase A), 5-mdC was mainly localized in the apical dome, leaf primordium and procambium (Fig. 2a-h), reaching 200 units of fluorescence intensity in the cells of the apical dome (tunica) (Fig. 3a and 3b). A lower 5-mdC signal was detected in the central cells of SAM (corpus), 50 units of fluorescence intensity (Fig. 3a and 3b). Apical dome, leaf primordium and procambium, the three areas of the bud with the most staining, were also strongly stained with DAPI indicating elevated number of cells and heterochromatization (Fig. 2c and 2d).

In phase B the distribution of nuclei with higher levels of methylation was reversed (Fig. 2i-p). The fluorescence signal was 100 units in the corpus while the meristematic apex showed intensities of only 50 units (Fig. 3c and 3d), although in the latter area there was a higher abundance of cell nuclei (Fig. 2k and 2o). In Fig. 2l, in addition to the terminal bud, there is an axillary bud which presents the tunica intensely marked similarly to the buds at the phase A.

**Figure 4:**
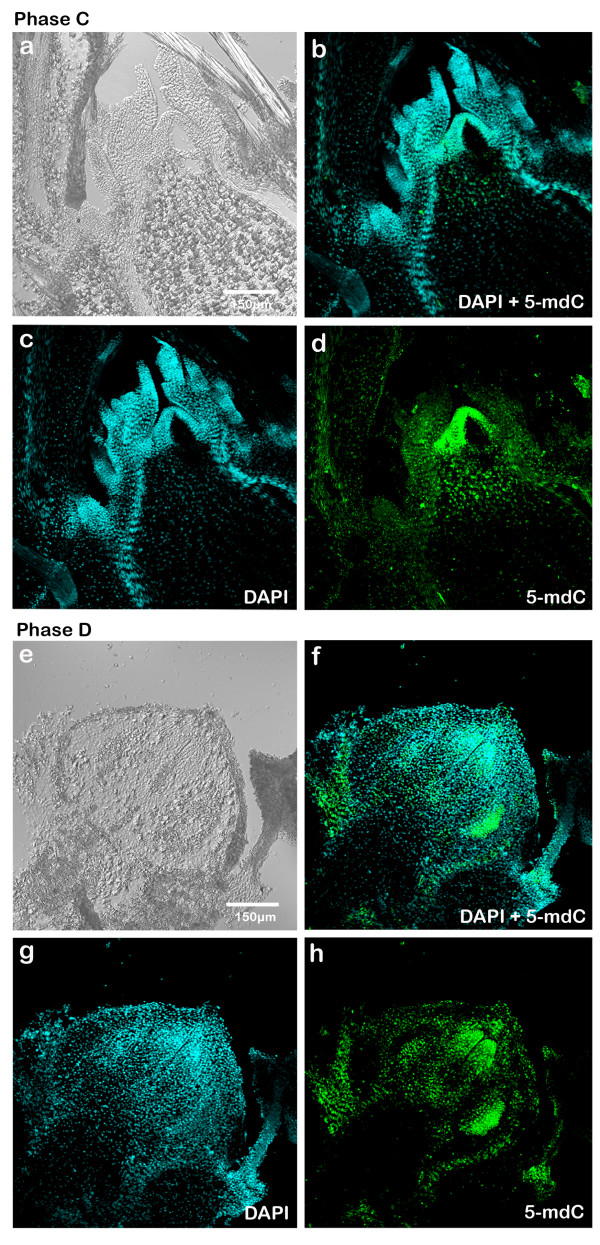
**Immunodetection of 5-mdC in sections of buds in phase C and D of development in longitudinal axis using a confocal microscope**. **(a) **Differential Interference Contrast (DIC) of bud in phase C (20×). **(b) **DAPI (blue signals) superposition and 5-mdC (green signals) of bud in phase C (20×). **(c) **DAPI labelling of nuclei of bud in phase C (20×). **(d) **5-mdC labelling of bud in phase C (20×). **(e) **DIC of bud in phase D (20×). **(f) **DAPI (blue signals) superposition and 5-mdC (green signals) of bud in phase D (20×). **(g) **DAPI labelling of nuclei of bud in phase D (20×). **(h) **5-mdC labelling of bud in phase D (20×).

Elongation of the bud was observed in phase C (Fig. 4a-d) with a very specialized leaf primordium in the apex. During this phase there was again preferred apical distribution in the signal of 5-mdC, showing an intense labeling in the leaf primordium, meristematic apex and procambium.

In phase D (Fig. 4e-h) the most intense fluorescence signal was mostly localized in the differentiated parts of the flower: sepals, petals and surrounding tissues, all of them also intensely stained with DAPI (Fig. 4f and 4g).

### Quantification of AcH4

**Figure 5:**
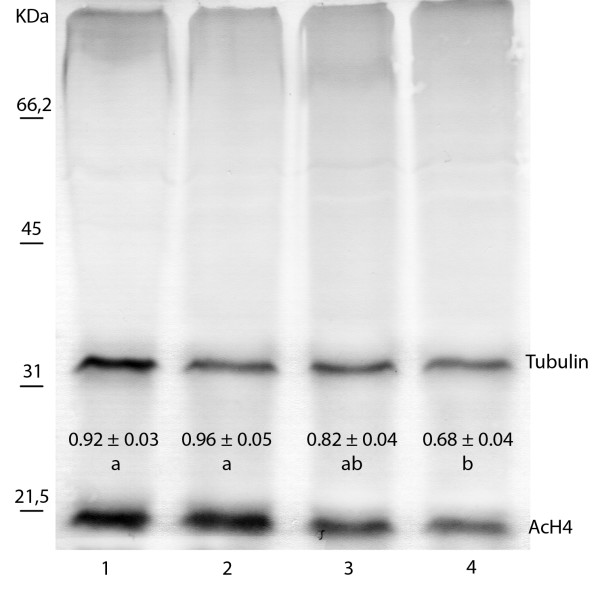
**Analysis of AcH4 and tubulin of buds in phase A, B, C and D by immunoblotting. (The figure shows a representative membrane)**. Average values of abundance index of AcH4 (± SE). Lane 1: tubulin and AcH4 of bud in phase A; Lane 2: tubulin and AcH4 of bud in phase B; Lane 3: tubulin and AcH4 of bud in phase C; Lane 4: tubulin and AcH4 of bud in phase D. Marker bands are indicated to the left of the blot. Different letters indicate significant differences between development phases of bud (One-way ANOVA; p ≤ 0,001; n = 4).

Protein extracts from the four development phases of buds described, revealed single bands of approximately 15 and 35 kDa for AcH4 and tubulin, respectively (Fig 5).

In phase A and B of the bud, vegetative growth and cellular reprogramming, higher levels of AcH4 were observed (0.92 ± 0.03 and 0.96 ± 0.05, respectively), decreasing in phase C (0.82 ± 0.04), corresponding to the rest stage, and reaching the lowest levels during phase D (0.68 ± 0.04) at the end of floral development of buds.

### AcH4 Immunolocation during floral development

**Figure 6:**
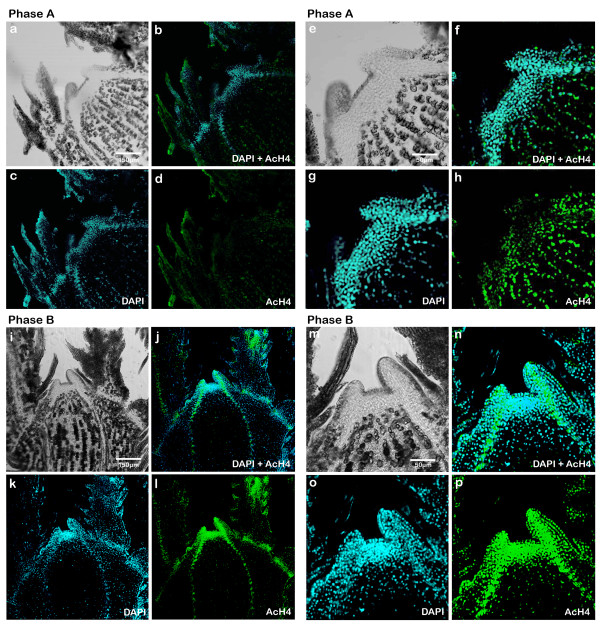
**Immunodetection of AcH4 in sections of buds in phase A and B of development in longitudinal axis using a confocal microscope**. **(a) **Differential Interference Contrast (DIC) of bud in phase A (10×). **(b) **DAPI (blue signals) superposition and AcH4 (green signals) of bud in phase A (10×). **(c) **DAPI labelling of nuclei of bud in phase A (10×). **(d) **AcH4 labelling of bud in phase A (10×). **(e) **DIC of bud in phase A (40×). **(f) **DAPI (blue signals) superposition and AcH4 (green signals) of bud in phase A (40×). **(g) **DAPI labelling of nuclei of bud in phase A (40×). **(h) **AcH4 labelling of bud in phase A (40×). **(i) **DIC of bud in phase B (10×). **(j) **DAPI (blue signals) superposition and AcH4 (green signals) of bud in phase B (10×). **(k) **DAPI labelling of nuclei of bud in phase B (10×). **(l) **AcH4 labelling of bud in phase B (10×). **(m) **DIC of bud in phase B (40×). **(n) **DAPI (blue signals) superposition and AcH4 (green signals) of bud in phase B (40×). **(o) **DAPI labelling of nuclei of bud in phase B (40×). **(p) **5-mdC labelling of bud in phase B (40×).

**Figure 7:**
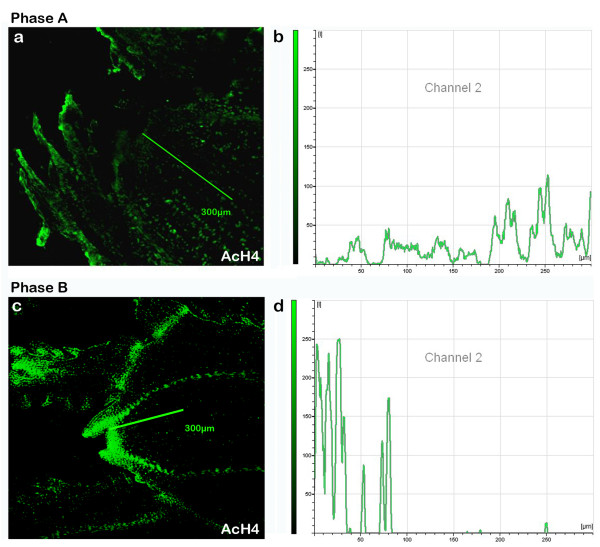
**Intensity of AcH4 fluorescence markers of buds in phase A and phase B**. **(a) **AcH4 labelling of bud in phase A (10×). **(b) **Intensity of AcH4 fluorescence markers along the line shown of bud in phase A **(c) **AcH4 labelling of bud in phase B (10×). **(d) **Intensity of AcH4 fluorescence markers along the line shown of bud in phase B.

The immunolocalization of AcH4 showed an opposite profile to 5-mdC and DAPI. In phase A (Fig. 6a-h), the AcH4 signal was lower in the apical dome, it increased gradually towards the meristem base reaching the maximum marker intensity (about 150 fluorescence units) in the central cells of SAM (corpus) (Fig. 7a and 7b).

In phase B (Fig. 6i-p), the distribution of nuclei with higher levels of acetylation was reversed, so that the fluorescence signal achieved was 250 units of intensity in the meristematic apex while in the corpus almost 50 units of intensity was detected (Fig. 7c and 7d).

**Figure 8:**
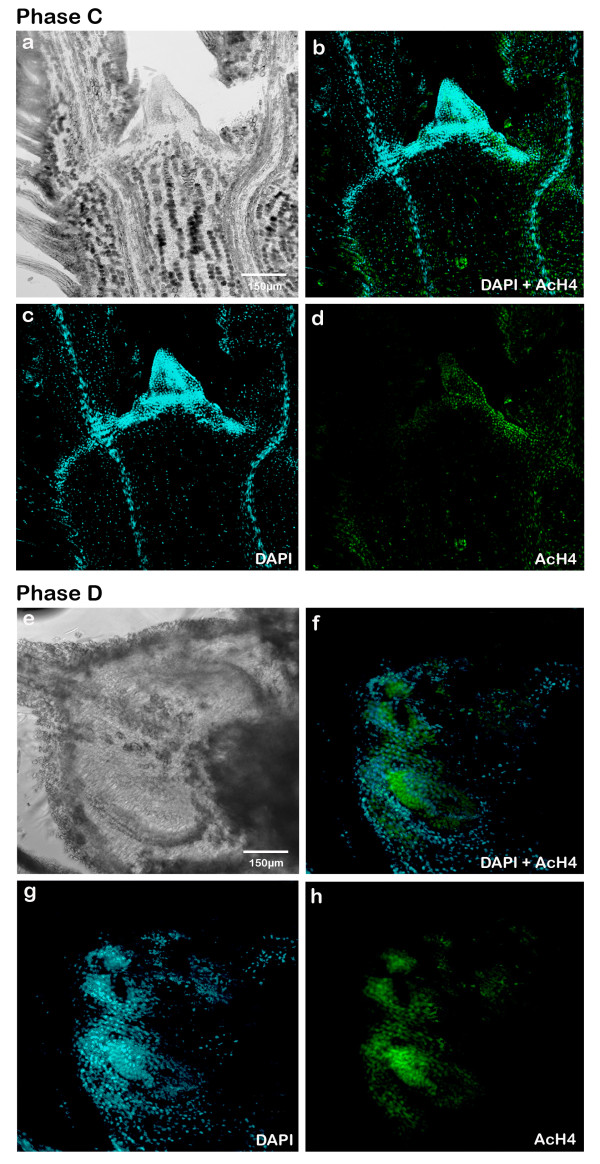
**Immunodetection of AcH4 in sections of buds in phase C and D of development in longitudinal axis using a confocal microscope**. **(a) **Differential Interference Contrast (DIC) of bud in phase C (20×). **(b) **DAPI (blue signals) superposition and AcH4 (green signals) of bud in phase C (20×). **(c) **DAPI labelling of nuclei of bud in phase C (20×). **(d) **AcH4 labelling of bud in phase C (20×). **(e) **DIC of bud in phase D (20×). **(f) **DAPI (blue signals) superposition and AcH4 (green signals) of bud in phase D (20×). **(g) **DAPI labelling of nuclei of bud in phase D (20×). **(h) **AcH4 labelling of bud in phase D (20×).

Also in the immunolocalization of AcH4 in phase C an elongation of the bud (Fig. 8a-d) and at the apex very specialized leaf primordium were observed. During this phase, the AcH4 signal was less intense, in general, all over the bud (Fig. 8a-d), in contrast to what happened in this phase with the immunodetection of 5-mdC.

Finally, in phase D (Fig. 8e-h) the AcH4 signal was very diffuse and decentralized, which indicated a high degree of cell differentiation and heterochromatinization.

The acetylation data are coherent with methylation changes also observed in these stages. Both mechanisms showed opposite profiles in every phase, indicating that H4 deacetylation and DNA methylation cooperate in restructuring chromatin during floral development in azalea.

## Discussion

The floral transition in azalea involves a series of changes at the molecular level that seem to be reflected in the levels of 5-mdC. The patterns of methylation observed were similar in both cultivars which validate the role of global DNA methylation as an indicator of development during floral transition in azalea [8]. However, there were some differences in global methylation between cultivars that could be due to their specific characteristics. Blaauw's Pink showed faster growth and a more robust architecture than Johanna. According to Hasbún et al. [28] the great morphogenic ability and growth of the apical meristems of juvenile individuals is associated with low levels of DNA methylation and higher levels of DNA methylation are related to the mature individuals with less morphogenic ability. Therefore the low level of global methylation in Blaauw's Pink may be associated with its higher morphogenetic ability that requires it to have a more dynamic metabolism and higher levels of gene expression. Berdasco et al. [29] recently described in *Arabidopsis *cell suspensions a chemically-induced 3% decrease in DNA methylation, that led to the change of the expression of 1794 sequences.

We have delimited in azalea four phases during the transition from vegetative to floral buds, the same number that defined by Bodson [2] based on phenological studies. However, the levels of methylation and the bud morphological observation permitted us to delimitate these phases (that we named phase A, phase B, phase C and phase D) with greater precision than Bodson [2]. Furthermore a stage of cell reprogramming (Fig 1), similar to what has been defined in other development processes both in plants and mammals [20,21], was identified.

Buds in phase A showed a level of methylation and morphology similar to that observed in a vegetative bud [8].

In the following stage, phase B, the most abrupt changes occur, nevertheless at macromorphological levels no changes can be detected, this phase seems to be the key stage during the floral transition in this species. In the first place a sudden fall in the percentage of global methylation is observed (8% 5-mdC), which seems to be related to the cellular reprogramming required to develop the new program of gene expression, in a way similar to what occurs in mammals [20,30]. During this phase, too, an increase in the levels of AcH4 is observed, confirming that this period corresponds with a stage of cell dedifferentation and euchromatinization. These epigenetic changes reduce the silencing of the target genes and increase cellular plasticity by facilitating the accessibility of transcription factors to developmental regulators and easing the switch to new epigenetic states [21]. Therefore the stage of cell differentiation is related to the rapid increase in the levels of methylation observed [21,31] and the initiation of the program of gene expression required for the differentiation of floral bud.

In the other two phases, C and D, DNA methylation and H4 acetylation are less variable, nevertheless the morphological changes are more obvious. The morphological study revealed that while in phase C, the bud seems be in rest, and in phase D floral development is completed. The high degree of floral differentiation of this stage (phase D) is accompanied by a high percentage of global methylation [12] and low H4 acetylation, which shows the relationship between DNA methylation, H4 deacetylation and differentiation in plants [8,32-34].

The transition from vegetative to reproductive development of the buds is accompanied by a number of changes in the physiology of the plant. These changes include an acceleration of cell division at the apex, elongation of the stem, and also the formation of flowers at the flanks of the shoot apical meristem. The transition from the vegetative to reproductive stage of development is controlled by multiple environmental and endogenous signals that ultimately modulate the expression of key gene regulators of floral identity: *APETALA1 *(*AP1*)/*CAULIFLOWER *(*CAL*) and *LEAFY *(*LFY*) [35,36]. Although the DNA methylation level and histone acetylation are important as global parameters descriptive of the state of development and gene expression [14,32], the differential spatio-temporal distribution of cells with a high 5-mdC signal and low AcH4 signal provides more detailed information since it permits the observation of the degree of differentiation and organization of the different cell types within the tissue [8,11,34]. In the floral transition of azalea, we observed that, besides the changes in the global DNA methylation and H4 histone acetylation, it was possible to observe a different distribution of 5-mdC and AcH4 according to the development of the bud, providing information about what types of cells are the most decisive at each stage of floral development.

Thus, in phase B of buds, it is possible to deduce great genomic activity, especially marked in cells of a more apical area of meristem, because of the low 5-mdC and high AcH4 signal observed. However, in the previous stage, phase A, the cells with higher morphogenic capacity are in the corpus, while the more apical cells of meristem responsible for the formation of leaf primordium present an intense labeling of 5-mdC and low AcH4. According to these results the central zone of the SAM in the vegetative stage can represent a stem-cell niche [37] which is activated after the floral induction for the developing floral bud [11]. In phase C, the apical meristem area shows again a greater degree of cell differentiation and heterochromatinization. Finally, in stage D groups of cells highly differentiated that appear to correspond to different parts of the flower were found.

The opposite distribution of 5-mdC and AcH4 observed in all phases of bud development, indicates a possible cooperation of both epigenetic mechanisms in chromatin remodeling [6,38] during the differentiation of floral buds in azalea. This ability to remodel chromatin organization, according to Costa and Shaw [39] may provide the basis for the plasticity in plant cell fate changes.

With these results it was possible to monitor the dynamics of bud development until the formation of the flower, establishing not only the relevance of cell localization within the meristem [37], but also the development phase of bud.

Floral induction is an essential stage led by environmental conditions and determinants the floral transition [5]. The exhaustive study of the floral induction would enable the effective handling of the timing of flowering, and the improvement of flowering in quantity and quality. Cellular reprogramming, marked by changes on the levels of global DNA methylation and H4 acetylation [20,21], as well as the loss of meristem zonation and the relocation of the cells with the higher morphogenic ability observed in phase B, have to be caused by the floral induction [11]. The molecular mechanisms which origin the reprogramming of meristem during floral induction are key to acquiring floral identity [35,36].

The transition from the vegetative to reproductive phase is a critical process in the life of plants. To achieve reproductive success, it is imperative that the plant has reached the level of energy and maturity that allows it to support the additional energy expenditure to maintain the reproductive organs. Therefore, the plants must integrate environmental and endogenous cues that regulate the onset of flowering. The plasticity that plants show in terms of their relationship with the environment and developmental programs reside in the signaling networks such as is described for the floral induction of *Arabidopsis *[5,35], which is largely regulated by the epigenetic code [6,38]. The ability of the epigenetic code to change rapidly and reversibly and its potential to remember of the changes made after cell division, suggests these mechanisms as candidates for the regulation of responses of plants to environmental conditions [18,32,33].

Chromatin states change during plant development. Cytologically defined heterochromatin increases during cell differentiation and organ maturation, while it decreases during cell dedifferentiation and reorganization processes [15,23]. In this study we demonstrate large-scale reorganization of chromatin by local changes in DNA methylation and histone H4 acetylation during floral transition of azalea.

## Conclusion

In conclusion, we can say that the complex process of floral transition is under epigenetic control and that the reorganization of chromatin seems to be decisive for coordinating floral development in azalea. The results of this work indicate that both DNA methylation and H4 deacetylation have particular dynamics during the transition from the vegetative to reproductive stage of development, and also the existence of an epigenetic reprogramming phase that is analogous to that in mammals is shown. Both epigenetic mechanisms act simultaneously and co-ordinately, restructuring the chromatin and regulating the gene expression in SAM development and floral differentiation.

## Methods

### Plant material

Four-year-old plants from two cultivars of azalea with different floral phenotypes (Blaauw's Pink double flowers and Johanna simple flowers), growth capacities (Blaauw's Pink high vegetative growth and Johanna low vegetative growth) and with known ability to bloom under long-day conditions were used for the analysis. Rooted cuttings of azalea were supplied by Fomento Vegetal, S.A. nursery (Asturias, Spain) in May 2003. The plants were grown outside in experimental fields belonging to SERIDA (Servicio Regional de Investigación y Desarrollo Agroalimentario de Asturias) in Villaviciosa (5°25' O, 43°28' N) (Spain) with a planting density of 3 plants per m^2 ^and fertirrigated three times a week with the following nutritional balances (N-P-K): 1-0.5-0.5 from May to July, 1-5-0 two weeks, 1-2-2 in August and 1-1.5-3 in September and October. Each spring the plants were transplanted into bigger pots containing pinus bank as substrate, supplemented with slow release fertilizers (3.5 g/L, Basacote Plus 6 M, Compo).

Every month, from July until February, apical buds were randomly taken from twenty different plants per cultivar (forty in total) grouped into four blocks of five plants. Samples were stored at -80°C until DNA and protein extractions were done.

For immunohistochemical and histological analysis only the Johanna cultivar was sampled and the buds were fixed immediately. For immunohistochemical analysis 4% paraformaldehyde in Phosphate-Buffered Saline 0.1 M, pH 7.3 (PBS) overnight at 4°C was used and, after that, they were stored at this temperature in 0.1% paraformaldehyde in the same buffer. For histological analysis glutardialdehyde in phosphate buffer 0.05 M, pH 5.8 at 4°C was used to fix and store the material.

### DNA extraction

Genomic DNA was extracted from 100 mg of buds (fresh weight) using a plant genomic DNA extraction kit (DNeasy Plant Mini, Qiagen) according to the manufacturer's instructions with modifications of Valledor et al [40]. To prepare completely RNA-free samples, 10 μL RNase A was added (Qiagen). Finally DNA was concentrated into DyNA Vap (Labnet) and resuspended in _dd_H_2_O to a concentration of 1 μg/μL. The samples were stored at 4°C until use.

### Global DNA methylation

For genomic DNA hydrolysis 5 μL DNA samples (0.5-2 μg μL^-1) ^were denatured by heating for 2 min and cooling rapidly on ice. Then, 0.25 μL of 10 mM ZnSO_4 _and 0.5 μL of nuclease P1 (Sigma; 200 U/mL in 30 mM C_2_H_3_O_2_Na) were added. Mixtures were incubated for 16 h at 37°C. Next, 0.5 μL Tris (0.5 M, pH 8.3) and 0.25 μL alkaline phosphatase [Sigma; 50 units/mL in 2.5 M (NH_4_)_2_SO_4_] were added and mixtures were incubated for an additional 2 h at 37°C. Hydrolyzed solutions were centrifuged for 20 min at 15000 g. Finally samples were analysed by HPCE (CIA, Waters Chromatography) according to Fraga et al. [41] with the modifications of Hasbún et al. [42].

Four biological and two analytical replicates per sample were taken. Quantification of the relative methylation of each DNA sample was performed as the percentage of 5-methyldeoxycytidine (5-mdC) peak of total deoxycytidines (dC + 5-mdC) peaks.

### Histological and Immunohistochemical studies

Glutardialdehyde fixed buds were dehydrated through an ascending ethanol series and included in liquefied paraffin. Samples were cut at 10-15 μm with a rotation microtome (Mod 1130/Biocut) and deparaffined in xylol. Then the sections were stained with 0.1% toluidine blue in phosphate buffer 0.1 M, pH 6.8. Finally the samples were visualized using an optical microscope (Nikon Eclipse E600).

Immunolocalization of 5-mdC and AcH4 was performed following the procedure described by Testillano and Risueño [43] with some modifications [44]. Paraformaldehyde fixed buds were cryosectioned at 30-40 μm thickness using a cryomicrotome Leica CH 1510-1 (Leica Instruments). Then the sections were permeabilized and treated with the antibodies. After the blocking reaction (5% bovine serum albumin in PBS, for 10 min), the cuts were incubated with anti-5-mdC mouse antibody (Eurogentec, Cat. no. BI-MECY-0100) diluted 1/50 in 1% blocking solution in one case and with anti-AcH4 rabbit antibody (Upstate, Cat. no. 06-866) diluted 1/25 in the other. Alexa Fluor 488-labelled anti-mouse polyclonal antibody (Molecular Probes, Cat. no. A-11001) diluted 1/25 was used as the secondary antibody for the 5-mdC detection and Alexa Fluor 488-labelled anti-rabbit polyclonal antibody (Molecular Probes, Cat. no. 11008) for the acetylated H4 detection. Finally the slides were counterstained with DAPI (4', 6-diamidino-2-phenylindole; Fluka).

In both immunochemical detection (5-mdC and AcH4) the negative controls were obtained replacing the primary antibody by PBS.

Fluorescence was visualized using a confocal microscope (Leica TCS-SP2-AOBS) connected to a workstation and the images were processed with Leica Software (LCS 2.5).

### Protein extraction and blot hybridization

Proteins were extracted from 250 mg of buds (fresh weight). After homogenization with liquid nitrogen using a mortar, the samples were incubated in extraction buffer 5× [100 mM Tris, pH 8.0, 10% SDS, 5% β-mercaptoethanol, 1 mM phenylmethylsulphonylfluoride (PMSF)] for 10 min at 95°C. Then, proteins were precipitated with 5 vol of acetone containing 0.07% Dithiothreitol (DTT) at -20°C. The pellet was resuspended in acetone and sonicated in an ultrasound bath (2 × 30 s), the precipitate was recovered after centrifugation at 15000 g for 20 min. The pellet was air-dried and solubilised in 1D buffer (8 M Urea, 2% CHAPS w/v, 8 mM DTT, 40% glycerol). Insoluble material was removed by centrifugation at 15000 g for 10 min. The protein concentration was determined using the Bradford assay (Bio-Rad) with ovoalbumin as standard.

Denaturalized proteins and standards (Low Range, Bio-RAD) were separated by electrophoresis (110 V) in 15% acrylamide SDS gels (Mini-PROTEAN II Multi-Casting Chamber; BIO-RAD) and then transferred to Immobilon membranes (Millipore Corp., Bedford, MA) by electroblotting (350 mA for 2 hours). For immunodetection, strips were blocked in a 2% dilution of powdered skimmed milk in PBS containing 0.05% Tween 20 at 4°C overnight. Then, membranes were incubated for 2 and 1 h with both primary and secondary polyclonal antibodies diluted 1/2000 and 1/1000, respectively, in the blocking solution. The primary antibodies used were anti-AcH4 polyclonal antibodies (Upstate, Cat. no. 06-866) and anti-delta-tubulin polyclonal antibodies (Chemicon, Cat. no. AB3203) as control of constitutive protein expression [45]. The secondary antibodies used were anti-rabbit IgG conjugated to alkaline phosphatase (Calbiochem, Cat. no. 401312).

Finally the strips were washed (0.2% powdered skimmed milk in PBS and 0.5% Tween 20) for 5 min and the secondary antibodies revealed by treatment with a nitroblue tetrazolium, bromo-chloro-indolyl-phosphate (NBT-BCIP) mixture. Densitometric measurements were taken after immunodetection using the Kodak.1D v 3.6 Scientific Imaging Systems. Abundance index was calculated as follows: AcH4 band intensity/delta-tubulin band intensity. Four biological replicates per sample were taken.

### Statistical analysis

Statistical analyses were performed with SigmaStat 3.1. software. For methylation data, a two-way ANOVA (cultivar and date) were used, followed by a Holm-Sidak test when significance differences were detected (α ≤ 0.05). For AcH4 data one-way ANOVA (development phase of bud) followed by a Holm-Sidak test (p ≤ 0.05) were applied.

## Authors' contributions

MM conceived, carried out the research and wrote the manuscript; IF contributed to carry out the research and supervised lab work; LV contributed to the development of the research methodology and to edit paper; RR participated in the design of the study and to initiate the research; MJ supervised the research and helped to draft the manuscript.

All authors have read and contributed to the writing of the manuscript.
